# Enhancing Psychiatry Residency Training: Restructuring Didactics and Morning Reports to Improve Resident Education and Engagement

**DOI:** 10.1192/bjo.2025.10252

**Published:** 2025-06-20

**Authors:** Lolwa Altheyab, Majid Alabdulla, Yousaf Iqbal, Sazgar Hamad, Pratyaksha Sinha

**Affiliations:** 1 Hamad Medical Corporation, Doha, Qatar; 2 Qatar University, Doha, Qatar

## Abstract

**Aims:** The psychiatry residency programme in Qatar recently faced challenges with didactic activities, prompting the formation of an educational committee composed of trainees and supervisors. Their mission was to revamp the curriculum to meet competency-based standards and address accreditation concerns. A pivotal change was designating one uninterrupted day per week for education. This structured day now starts with a redesigned morning report focused on real-time discussions of overnight emergency cases, integrating various disciplines to broaden clinical insight. Following this, residents engage in newly established, two-hour resident-led sessions covering DSM–5 diagnoses, multiple-choice question (MCQ) practice, simulated learning, and psychiatric disorder management. Afterward, junior residents join Balint groups for reflective practice, while senior residents participate in psychotherapy supervision. Additional activities include case conferences, journal clubs, and workshops, culminating in didactic lectures led by senior clinicians.

**Methods:** To evaluate the impact of this revamped schedule, the committee conducted a cross-sectional, anonymized survey among all psychiatry residents. The survey contained six core questions, each rated on a five-point Likert scale (1=strongly disagree to 5=strongly agree), supplemented by a free-text section to gather qualitative feedback. Of the 30 residents invited to participate, 22 (73%) completed the survey. Quantitative data were then analysed to assess perceived benefits, identify challenges, and guide further adjustments.

**Results:** Results indicated that first- and second-year residents reported substantial gains from both the structured teaching and the opportunity to collaborate closely with peers. In contrast, third- and fourth-year residents suggested splitting certain sessions by training level to better match their advanced needs. Another noted concern was the level of faculty engagement, with respondents requesting increased direct supervision, more robust involvement in lectures and case discussions, and pre-assigned case presentations. Residents also expressed a desire for more frequent mock interviews and for simulated sessions in additional languages, targeting the diverse patient population seen in Qatar. Dedicated sessions for MCQ practice were requested to enhance board exam preparation.

**Conclusion:** Overall, these curricular changes have produced a more interactive and clinically relevant educational experience. However, the survey highlighted clear areas for improvement, particularly with regard to tailoring sessions to various training levels and expanding faculty participation. Further refinements will rely on continuous resident feedback, systematic assessment of learning outcomes, and enhanced faculty development. By maintaining an adaptive and trainee-centred approach, the programme aims to strengthen psychiatric education and foster the clinical competencies necessary for high-quality patient care.

